# Implementation of opioid maintenance treatment in prisons in North Rhine-Westphalia, Germany – a top down approach

**DOI:** 10.1186/s13011-020-00262-w

**Published:** 2020-03-10

**Authors:** Kathrin Böhmer, Henrike Schecke, Irmgard Render, Norbert Scherbaum

**Affiliations:** 1 Department of Addictive Behaviour and Addiction Medicine, LVR-Hospital Essen, University of Duisburg-Essen, Virchowstr. 174, 45 147 Essen, Germany; 2 Ministry of Justice of North-Rhine Westphalia, Düsseldorf, Germany

**Keywords:** Opioid maintenance treatment, OMT, Prison, Germany

## Abstract

**Background:**

Opioid Maintenance Treatment (OMT) is a well-evaluated treatment of opioid use disorder (OUD). Especially, under the condition of imprisonment, OMT is a preventive measure regarding infectious diseases such as hepatitis C. However, only a minority of prisoners with OUD are currently in OMT in numerous countries. In 2009, the Ministry of Justice of the federal state of North Rhine-Westphalia (NRW), Germany, launched the process of implementing OMT in prisons with various elements (e.g. development of recommendations regarding the treatment of prisoners with OUD, monitoring the number of prisoners in OMT, education of prison doctors). In the recommendations OMT was defined as the gold standard of treatment of OUD.

**Methods:**

To assess the effectiveness of the implementation strategy a survey on the prevalence of OMT in prisons in NRW was carried out twice a year by the Ministry of Justice between 2008 and 2016. Participants were prisoners in NRW, Germany. The diagnosis of OUD at admission to prison and the treatment state on survey dates was measured.

**Results:**

The number of prisoners in NRW dropped from 17,301 in 2008 to 16,432 in 2016. In the same period, the number of prisoners with OUD (mainly males) dropped from 4201 persons to 3650 persons and the number of prisoners in OMT increased from 139 persons (3.3%) to 1415 (38.7%) persons.

**Discussion:**

Currently, the percentage of prisoners with OUD in OMT in NRW is almost reaching the treatment rate outside prisons in Germany (45–50%). However, after release from prison there is still a high risk for a discontinuation of OMT.

**Conclusions:**

Overall, the top-down approach of implementing OMT in prisons in the federal state of NRW was effective.

## Background

Substance use disorder is one of the leading problems in the international field of health-care [1]. An estimated quarter of a billion people, around 5% of the global adult population, used drugs at least once in 2015 and of those drug users 29.5 million, 0.6% of the global adult population, suffered from a drug use disorder [2]. Opioid use disorder (OUD) in particular is associated with a high mortality rate and a high burden of disease [3]. The number of people with OUD in Germany has been estimated about 146,580–174,064 persons [4]. After several years of decrease, the number of drug-related deaths (DRD) in Germany has increased again since 2012 to 1272 DRDs in 2017 [5].

According to current concepts, OUD is a disorder with a chronic course [6]. Abstinence-oriented treatment has only a limited success in the treatment of OUD. Therefore opioid maintenance treatment (OMT) has been established. Its major aim, the reduction of the use of illegally acquired heroin, is well proven [7]. In addition, OMT lowers the risk of the transmission of infectious diseases such as hepatitis C or HIV by reducing needle sharing; patients in OMT report a higher well-being and a better social functioning; the rate of drug-related crimes decreases for patients in OMT; patients in OMT have less stress because the pressure to gain drugs is substantially reduced; and patients in OMT were found to have reduced mental and physical comorbidities [8–11]. Hence, OMT has become the gold standard of treatment of patients with OUD in many countries. In 1991, OMT was legally accepted as a treatment in Germany and its costs are covered by the statutory health insurances [12]. The number of patients in OMT in the community increased from approximately 52,700 in 2003, to 74,600 in 2009 up to 78,800 patients in OMT in 2017 [13]. Currently, the rate of people affected by OUD in maintenance treatment is about 45–50% in Germany.

Many prisoners suffer from OUD and compared to the community, people, who use drugs are even over-represented in prisons [14]. According to estimates, 30% of all male prisoners and 50% of all female prisoners in Germany inject drugs [15]. Despite rigid controls, drug consumption occurs in prisons [16]. The condition of imprisonment favours risky behaviours due to concentrated at-risk populations and risk-conducive conditions such as violence or overcrowding [17]. In an Irish prison population, significantly more needle sharing was practiced during imprisonment compared to the month before imprisonment [18]. Due to the high prevalence of OUD among prisoners and the specific health risks in this environment OMT for prisoners is recommended. However, in numerous states a systematic implementation of OMT in prisons is still lacking [19]. OMT in prison settings has basically the same aims and effectiveness as OMT in the community [20], particularly preventing the spread of hepatitis C and HIV-infection due to reduced heroin injecting and needle sharing; reduction of violent behaviour in prisoners with a substance use disorder [21]; reduction of heroin use during imprisonment; reduction of the risk of dying immediately after release due to an overdose and increasing the chance of continue OMT upon release [22, 23].

The World Health Organization recommends OMT in prisons as standard treatment [24]. In Germany, the costs of the medical treatment of prisoners are covered by the ministry of justice of the respective federal state during imprisonment. According to the principle of equivalence prisoners have a legal entitlement to receive the treatment during imprisonment which would be covered by statutory health insurances, if the persons were outside prison. This principle is also integrated in article 40.2 in the “European Prison Rules” [25]. Whereas the principle of equivalence seems to be effective in the treatment of e.g. diabetes mellitus and hypertension, only a minority of people with OUD in German prisons have been in OMT for the last decade, although no official statistics are available regarding this topic. According to the German Prison Act in 2006, the 16 federal states of Germany have the legislative competence of the penal system [26]. Therefore, all federal states are independently responsible for providing adequate medical care (including OMT) for prisoners.

North Rhine-Westphalia (NRW) is the most populous federal state of Germany with about 18 million inhabitants. Given the low rate of OMT in German prisons, the Ministry of Justice of the federal state NRW launched the process of implementation in 2009. It has several elements:

Development of recommendations for the treatment of prisoners with OUD: The recommendations were developed by a task force consisting of representatives of the boards of physicians of North Rhine and of Westphalia, respectively, directors of prison hospitals in NRW, prison doctors, and representatives of the Ministry of Justice NRW. The development of the recommendations was based on the guidelines of the German Board of Physicians [27], which explicitly point out the possibility of OMT in prisons. The target group of the recommendations were prison doctors as well as prison directors, who are responsible for the financial and personnel resources. The recommendations lay importance on the following aspects: OUD requires treatment. Since OUD continues during imprisonment, treatment has to be established or continued during imprisonment. OMT is a standard and well proven treatment, therefore it should be also carried out in prison according to the principle of equivalence. The recommendations entered into force as from January 15th, 2010 [28]. It was a strong statement of the Ministry of Justice that it expects that OMT is carried out in prisons. The recommendations were elaborated in a mixed group including three prison doctors to strengthen its impact on the group of prison doctors.

Education: As a specific medical qualification is required in Germany to carry out OMT (“Suchtmedizinische Grundversorgung” meaning “basic care for substance use disorders”), prison doctors were asked by the Ministry of Justice to achieve this qualification by completing a course of 50 h. In addition, the recommendations were presented and discussed at a mandatory workshop for all prison doctors in 2010.

Monitoring: In principle, physicians in the community and in prisons are free in the choice of a treatment for an individual patient in Germany. In consequence, there are no directions of the Ministry of Justice or the prison directors to prison doctors how to treat an individual prisoner. Therefore, after the announcement of the recommendations there was no routine ministerial check at the level of individual prisoners with OUD, why they do not receive OMT. Rather a monitoring by the Ministry of Justice was established regarding the number of prisoners with OUD and the number of prisoners in OMT. These figures had to be reported by each prison twice yearly. In this way, the rate of prisoners in OMT was regarded as a criterion for the quality of care for people with OUD in a given prison. The data were presented and discussed at the mandatory meetings of prison doctors organized by the Ministry of Justice once yearly.

As the recommendations evaluate OMT as the gold standard of the treatment of OUD, there is a lack of quality of care if only single individuals with OUD in a prison are in OMT. This evaluation does not interfere with the right of physicians to state the indication for OMT for an individual person. In addition, after the announcement of the recommendations the prison doctor could be asked in case that an individual prisoner complains about not receiving OMT, why he did not carry out the gold standard treatment in this case.

The aim of this study is to assess the effectiveness of the implementation strategy for OMT in prison. We hypothesized that the number of prisoners with OUD in OMT would increase during the monitored period.

## Methods

In the federal state of NRW, Germany, there are 62 prisons with up to 18,500 prisoners located. In order to assess the effect of the described implementation process (and as part of this process), a survey was carried out twice a year (April and October) over the years of 2008–2016. The survey gathered data on the number of prisoners at the respective dates over all prisons in NRW, the number of prisoners with OUD, and the number of prisoners currently in OMT (methadone or buprenorphine). The twice-yearly report was carried out by different staff members of the participating prisons, e.g. prison doctors, social workers, or other professionals of the medical service. Data were centrally gathered and analysed at the Ministry of Justice NRW. The respective prison doctor diagnosed OUD based on a diagnostic interview held at the admission to prison. In Germany, OUD is diagnosed according to the diagnostic criteria of the ICD-10. In addition, a drug urine screen was carried out in order to detect use of heroin and of other substances (i.e. methadone, cocaine, cannabis, amphetamine, and benzodiazepine). Since 2017, a nationwide survey on OMT in prisons in Germany has been established which replaces the former survey just in NRW with slightly different methods.

As a part of OMT in prison, psychosocial interventions are offered. These differ between the participating prisons. However, every institution provides counselling for OUD, where prisoners can obtain information or advice if required.

## Results

The number of prisoners in NRW dropped from 17,301 (in 2008) to 15,781 (in 2017) at the qualifying dates for the survey. There were statistically significantly fewer prisoners in 2017 compared to 2008. A chi-square test of goodness of fit was performed to determine whether the number of prisoners was equally distributed between the years. The number of prisoners was not equally distributed, χ^2^ (1, *N* = 33,082) = 69.84, *p* < .01. At the respective dates, the number of prisoners with OUD also decreased significantly from 4201 in 2008 to 3650 in 2016 (χ^2^ (1, *N* = 7851) = 38.67, *p* < .01). In the period from 2008 to 2016, the number of prisoners in OMT increased from 139 persons out of 4201 prisoners with OUD (OMT rate: 3.3%) to 1415 out of 3650 prisoners with OUD (OMT rate: 38.8%). In 2017 the number of prisoners in OMT increased even further to 1603 (see Table 1). In 2016, there were significantly more prisoners with OUD in OMT than in 2011. A chi-square test of independence was performed to examine if OMT increased over time after the recommendations came into effect. The year 2011 was compared to 2016. As reference year, 2011 was chosen because it was the year after the recommendations of OMT in prison had entered into force and 2016 was chosen because it contained the most recent data. The relation between these variables was significant, χ^2^ (1, *N* = 8391) = 168.87, *p* = <.01.

**Table 1:** Development of prisoners undergoing Opioid Maintenance Treatment (OMT)

Date	Total number of prisoners at year/cutoff-date	Number of prisoners with opioid use disorder	Number of prisoners in opioid maintenance treatment	Proportion of prisoners with opioid use disorder in opioid maintenance treatment (in %)
2008	17,301	4201	139	3
2009	17,124	n/a	n/a	n/a
Oct., 2010	16,828	4701	751	16
Apr., 2011	17,263	4968	1035	21
Oct., 2011	17,021	4741	1209	26
Apr., 2012	17,410	4722	1297	27
Oct., 2012	17,203	4385	1380	31
Apr., 2013	17,003	4355	1374	32
Oct., 2013	16,710	4294	1413	33
Apr., 2014	16,575	4159	1441	35
Oct., 2014	15,954	3900	1493	38
Apr., 2015	15,926	3905	1500	38
Oct., 2015	15,640	3693	1442	39
Apr., 2016	16,432	3650	1415	39
Oct., 2017	15,781	n/a	1603	n/a

*n/a* No data were collected in 2009. In 2017, a nationwide survey was used. Since this version differed slightly from the years 2009–2016, the number of prisoners with opioid use disorder is unknown for 2017

**Fig. 1:**
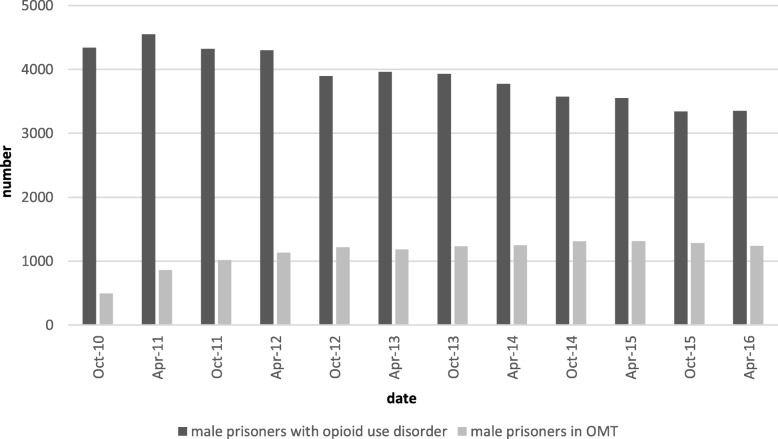
The proportion of male prisoners with opioid use disorder in opioid maintenance treatment

**Fig. 2:**
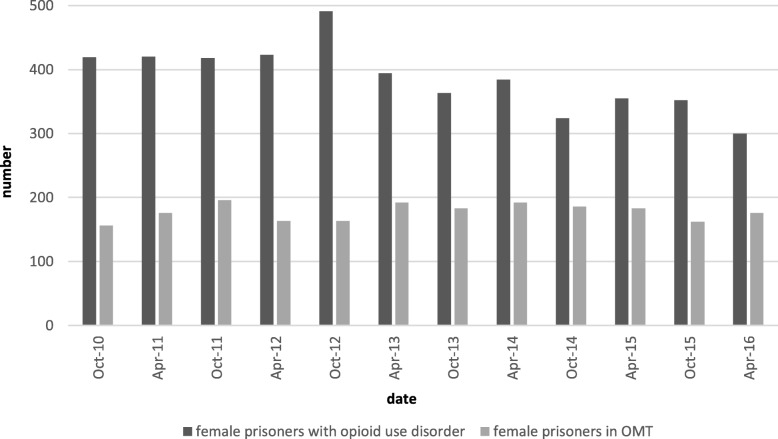
The proportion of female prisoners with opioid use disorder in opioid maintenance treatment

Prisoners with OUD were mainly male [in 2016: 3350 males (91.8%) and 300 females (8.2%)]. The proportion of male prisoners in OMT increased over the years from 11.4% in 2010 to 37.0% in 2016 (see Fig. 1). The proportion of female prisoners in OMT was already relatively high in 2010 with 37.2% (see Fig. 2). The number of female prisoners in OMT was stable during the observation period, although the total number of female prisoners with OUD decreased. Therefore, the proportion of female prisoners with OUD in OMT increased up to 58.7% in 2016 (see Fig. 2).

## Discussion

The data emphasize that the process of implementation of OMT in prisons in NRW was very effective. Currently, the percentage of prisoners with OUD in OMT in the federal state of NRW (38.7%) is almost reaching the treatment rate (about 45–50%) in the community in Germany. The effectiveness of the implementation process is based on several elements: the clear statement of the Ministry of Justice that OMT has to be implemented in prisons, treatment recommendations developed by the medical profession defining a standard of care, medical education of prison doctors regarding substance use disorders, and a monitoring system about the implementation of OMT.

Furthermore, the number of prisoners in NRW dropped from 17,301 to 15,781 on the qualifying survey dates. At the same time, the number of prisoners with OUD decreased from 4201 to 3650. An explanation for a reduction of prisoners with OUD in the studied time period might be a reduction of heroin-related offences in Germany. The overall number of drug-related offences increased from 231,007 in 2010 to 352,320 in 2018, whereas drug-related offences with relation to heroin decreased from 24,574 in 2010 to 11,402 in 2018. Since drug offences are among the main offences of people with OUD, this might be a partial explanation for a reduction of prisoners with OUD [29]. The number of people with OUD in the German population has been rather stable over the last 20 years in Germany [30].

Furthermore, in 2012, the Federal Cabinet adopted the National Strategy on Drug and Addiction Policy in Germany. The strategy aims to help individuals avoid or reduce the consumption of substances and is based on prevention, treatment, harm reduction measures, and repression [31]. The adoption of this new strategy might have had an influence on reducing the number of prisoners with OUD.

We also found a difference between male and female prisoners in OMT. The proportion of male prisoners in OMT increased over the years from 11.4 to 37.0%. The proportion of female prisoners in OMT was already relatively high in 2010 with 37.2% and remained rather stable during the following observation period.

A nationwide survey in German prisons found that female prisoners use opioids more often than male prisoners and that 34% of female prisoners had OUD compared to 19% of male prisoners [30]. Furthermore, this study confirms that more female prisoners were in OMT in prison: in 2018, 21.4% of male prisoners were in OMT and 53.6% of female prisoners were in OMT. Since our results for the federal state of NRW are in line with the results of the nationwide survey, they do not represent a specific effect for this particular federal state. A study of OUD in the German population estimated that 62.02% of females with OUD and 54.96% of males with OUD are in OMT [32], showing that females with OUD are generally more often in OMT.

Despite the effectiveness of the process of implementation, the majority of prisoners with OUD in NRW are still not in OMT. However, about half of the people with OUD in the community are also not in OMT in Germany. There may be different reasons for patients staying out of OMT. Firstly, OMT is a difficult choice for patients, who might fear treatment rules [9, 33]: Drug urine screens regarding not only heroin, but also possibly concomitantly used drugs are a treatment requirement in OMT in Germany. Concomitant use of other drugs can endanger the continuation of OMT in the community as well as in prison. Other requirements include a medically supervised administration of the daily dose of the maintenance medication and reliable compliance attending appointments with the treating physician and the social workers. For some patients this might seem impossible to achieve. Negative experiences with treatment, like side-effects such as sedation under methadone, episodes of OMT without success, as well as a lack of information may be additional reasons for staying out of OMT [17, 33]. In addition, patients might fear to become unable to withdraw from the maintenance medication [34]. Furthermore, prison inmates might wish to use the time in prison to get abstinent from all substances and might fear the long-term nature of OMT [35].

Moreover, the role of stigmatization for the implementation of OMT in prison should be emphasized. Even though OMT has been officially accepted as the gold standard treatment of OUD, it continues to be stigmatized. 78% of a study sample of patients currently in OMT reported having experienced stigma related to OMT [36]. Some people believe that people with OUD use OMT to get high, that OMT patients are incompetent, unfit to work and untrustworthy, and that people with OUD simply have a lack of willpower to overcome their disorder. In sum, a poor understanding of OUD as a chronic, relapsing disease is common. One explanation for the low implementation of OMT in prisons might be that stigmatizing attitudes could be also common in prison doctors, directors and other persons involved in planning and carrying out health care in prisons. In this context, it is noteworthy, that in the surveys at the qualifying dates the maintenance rates between the prisons varied largely. For example in April 2015, the rates were between 12 and 80% (only analysing prisons with at least 20 prisoners with the known diagnosis of OUD). Also of interest is the fact, that in order to receive OMT, 40 prisoners in Würzburg (federal state of Bavaria) went on a hunger strike in July 2016. The hunger strike ended unsuccessfully after 11 days [26]. The European Court of Human Rights stressed in its decision of September 1st, 2016 in a case against the Federal Republic of Germany the principle of equivalence in the care of prisoners suffering from OUD [37].

Even though the number of prisoners in OMT increased during the last years in NRW, there is the problem of continuity of OMT. This might regard the continuation of OMT at admission to prison as well as upon prison release. Besides the fact that an abrupt ending of OMT at imprisonment clearly offends against the legal principle of equivalence, this can be physically and psychologically distressing for the imprisoned patients [38] and even dangerous, since withdrawal symptoms have been found to be a trigger for suicide during the first week of prison [23]. In addition, the continuity of OMT in prison and after release is of high importance: Because of intervals of abstinence during imprisonment, the tolerance for drugs is reduced, meaning that a smaller dose can already result in life-threatening conditions [39]. A review by Hedrich et al. [20] revealed that pre-release OMT was highly associated with an increase in both, treatment uptake and retention after release. Sponsored by the Ministry of Health NRW, there is now an ongoing study to improve continuation of care, especially OMT, in the community after release from prison.

The following limitation of the study has to be discussed: The diagnosis of OUD is based on the diagnostic interview carried out by the prison doctor at admission of a prisoner to the prison. In addition, a drug urine screen is carried out in order to detect use of heroin and of other drugs. As the diagnostic criteria of OUD are mainly based on information given by the prisoner there might the risk of underdiagnosing OUD. However, the rates of prisoners with OUD out of all prisoners in NRW are within the range known from other federal countries in Germany [40]. In addition, there is no doubt that the rate of prisoners in OMT increased substantially in the observation period.

## Conclusions

Overall, our work has led us to conclude that the top-down approach of implementing OMT in prisons in the federal state of NRW was effective. It seems that the clear statement of the Ministry of Justice that OMT has to be implemented in prisons as well as treatment recommendations developed by the medical profession defining a standard of care, medical education of prison doctors and a monitoring system were important parts in increasing the amount of prisoners in OMT. The percentage of prisoners with OUD in OMT in NRW has increased continuously since 2009 and is now almost reaching the treatment rate in the community in Germany. Nevertheless, the majority of prisoners with OUD are not in OMT. Reasons for staying out of OMT might be fear of treatment rules, negative experiences with treatment or stigmatization. Furthermore, there is still a high risk of a discontinuity of OMT at admission to prison as well as upon prison release. Future research needs to focus on effective ways to ensure a consistent continuation of treatment in terms of the principle of equivalence.

## Data Availability

All data generated or analysed during this study are included in this published article.

## Abbreviations

DRD: Drug-related deaths
NRW: North Rhine-Westphalia
OMT: Opioid maintenance therapy
OUD: Opioid use disorder
